# A Food Retail-Based Intervention on Food Security and Consumption

**DOI:** 10.3390/ijerph10083325

**Published:** 2013-08-05

**Authors:** Richard C. Sadler, Jason A. Gilliland, Godwin Arku

**Affiliations:** Department of Geography, University of Western Ontario, 1151 Richmond Street, London, ON N6A 5C2, Canada; E-Mails: jgillila@uwo.ca (J.A.G.); garku@uwo.ca (G.A.)

**Keywords:** built environment, food access, food consumption, food security, Flint, Michigan

## Abstract

The effect of the built environment on diet (and ensuing health outcomes) is less understood than the effect of diet on obesity. Natural experiments are increasingly advocated in place of cross-sectional studies unable to suggest causality. The central research question of this paper, therefore, asks whether a neighborhood-level food retail intervention will affect dietary habits or food security. The intervention did not have a significant impact on fruit and vegetable consumption, and the intervention population actually purchased prepared meals more frequently. More problematic, only 8% of respondents overall regularly consumed enough fruits and vegetables, and 34% were food insecure. Further complicating this public health issue, the new grocery store closed after 17 months of operation. Results indicate that geographic access to food is only one element of malnutrition, and that multi-pronged dietary interventions may be more effective. The economic failure of the store also suggests the importance of non-retail interventions to combat malnutrition.

## 1. Introduction

The built environment is thought to influence behavior such that dietary habits may be constrained for those unable to access nutritious foods, an issue commonly called ‘food deserts’ in academic and public discourse. While the influence of some aspects of the built environment is well-defined, the causality of this pathway is less clear. Using survey data from before and after a food retail intervention in socioeconomically disadvantaged neighborhoods in Flint, Michigan, this research will answer: what is the relationship between a new grocery store and food consumption/food security? Statistical tests are run to determine whether this intervention had a significant impact, and recommendations are made based on the efficacy. Literature includes the various pathways from the built environment to malnutrition and the growth of scientific evidence on this subject.

### 1.1. Health and the Built Environment

Public health issues arising from unhealthy behaviors play a destructive role in societal well-being. In Canada and the United States, rates of adult obesity exceed 20%; comorbidities include high blood pressure, asthma, arthritis, and diabetes [1]. Although obesity is caused by a web of biological, environmental, and behavioral factors, one important causative agent includes health-degrading activities such as unhealthy eating and physical inactivity. In turn, habits of unhealthy eating can be exacerbated by the prevalence of inexpensive, unhealthy food [2].

The increase in diseases partly attributable to poor diet and lack of exercise—which also includes cardiovascular disease, heart disease, cancer, and musculoskeletal disorders—has an economic impact as well [3]. In the United States, annual health-care and prescription costs for patients coping with diet- and obesity-related diseases are 36% and 105% higher, respectively, than for average citizens [4]. The effects of diet-induced obesity are also manifest through decreased worker productivity, increased disability payments, and increased transportation costs [4]. Inadequate access to nutritious foods due to the design of the human-constructed built environment is now thought to contribute to this class of diseases by exacerbating malnutrition (in this sense, referring to a diet which lacks adequate nutritional quality for healthy functioning).

Recognizing that the design of the built environment influences behavior and, ultimately, public health outcomes, many social scientists now employ an ecologic framework for population health to attempt to explain the chain of causality [5]. Research using this framework suggests that income inequality and community social capital can play crucial roles in shaping health [6] and health-related ailments such as obesity [7]. As well, built environments with a high concentration of unhealthy food offerings can exacerbate the propensity for low-income consumers to develop health problems [6]. There is growing interest, therefore, in the relationship between food environments and health outcomes. The body of work on food environments is diverse, and considers various geographic, economic, and social factors as primary agents of behavioral change. Their methods are also increasingly sophisticated, as discussed below.

### 1.2. Food Environments and Diet/Diet-Related Disease

Research on socioeconomic inequality and food access often suggests that residents experience food environments in very different ways, especially when considering social and economic differences. While *systematic* inequalities do not exist uniformly from one region to another, some disadvantaged neighborhoods do have poor access and contain a disproportionate share of residents with poor dietary habits [8]. These inequalities and differences in research findings can be found in many diverse communities. Two studies from Melbourne, Australia, demonstrate that neighborhoods with affluent residents have better accessibility to nutritious foods [9,10]. Another study in Edmonton, Canada, indicates that *disadvantaged* neighborhoods have better access to nutritious foods [11]. A metropolitan-wide study in Minneapolis, Minnesota, showed that prices of healthy foods were less expensive in disadvantaged neighborhoods [12]. Yet other studies in the United States and the United Kingdom (UK) suggest that disadvantaged residents can access inexpensive, *un*healthy foods better than residents in other neighborhoods [13,14]. Whether these inequalities in price or availability of food translate into differences in health outcomes is also a subject of considerable debate.

Researchers have examined these relationships between food environments and health outcomes. A study in Leeds, UK, found a positive relationship between density of fast food around residences and obesity of children [14]. Similar results on adult obesity were found in two counties in North Carolina and Mississippi, including a negative relationship between the density of supermarkets and adult obesity rates [15]. A nationwide study of New Zealand census mesh blocks found that access to fast food was not associated with prevalence of overweight adults—in fact, those most distant from fast food were more likely to be overweight [16]. These examples are representative of the wide range of results found in studies of access to food and health outcomes such as obesity, suggesting that food environments do not always have significant effects on populations. Even so, the effects of social inequality can be compounded where gaps do remain in food environments [17], and a substantial discourse has emerged from this concern.

### 1.3. “Food Desert” Discourse

Practitioners and academics frequently allude to the concept of ‘food deserts’ to describe these socioeconomically disadvantaged areas with poor access to nutritious foods. The concept is an increasingly popular subject of public interest and debate, and public programs have been deployed to study the location and existence of these phenomena. The Let’s Move Campaign’s Healthy Food Financing Initiative has worked with large retailers and communities to secure commitments to build stores in food deserts [18]. The USDA recently created a nation-wide food desert locator to bring awareness to the issue and help guide retail-based interventions, but the locator is plagued by poor data quality [19]. This is but one example of public policy based on inaccurate descriptions of food deserts. Consequently, academics have been critical of using this term without carefully contextualizing the issue.

Bedore suggests that beyond viewing food deserts as static geographic entities irrespective of societal context, they should be understood as “part of a historical continuum of capitalist urbanism” (p. 2) [20]. She problematizes the concept by indicating that many methodological and conceptual debates exist in defining food deserts:

“…declining food access can be understood critically through an analysis of the time- and place-specific nature of capital, including its uneven penetration in local economies, the extent of its concentration and consolidation, and its impact on the local built environment”.

(p. 2) [20]

Donald likewise notes that the term ‘food deserts’ is highly contested among researchers, and that: “Many reject the image of a bleak and desolate urban landscape while others hope to dispense altogether with a term that has potentially racialized implications linking people of colour to barren environments” (p. 2) [21].

Donald and Bedore are not alone in their critiques of the concept of food deserts. There is a lack of consensus on the definition, which can result in inconsistency or uncertainty when devising public policy [22]. Some have suggested abandoning the term altogether in favor of more general terms such as access [23]. Other researchers suggest that individual-level circumstances such as mobility are a better indicator of difficulties with food access than geographic metrics such as the lack of a grocery store in a community [24]. Increasing the use of psychometric measures (including perceptions, beliefs, and attitudes of consumers) may help to more accurately identify the factors involved in healthy eating behavior [5].

Although disagreement remains in discourse and literature on the extent to which food deserts influence diet or health, living in such an area is at least likely to exacerbate the potential for dietary problems related to poor access to nutritious foods, especially for those with mobility issues. Residents living in communities with an abundance of so-called ‘food deserts’ are also likely more disadvantaged in terms of lack of access to employment, health services, and education, because it is often these communities that have been affected most by a changing world economy. Thus food deserts should be recognized as a by-product of many social issues, and not merely as geographic gaps in the food environment [20]. A thorough engagement with community-specific assessment and neighborhood identification is important for practitioners, therefore, to devise and advocate for interventions to improve healthy eating behaviors. These interventions should also aim toward evaluation designs which can help suggest causality in the food environment.

### 1.4. Innovative Study Designs in Food Environments Research

Most ecologic studies of food environments and socioeconomic factors or health-related outcomes have been based on observational or cross-sectional methods. Cross-sectional research is popular among social scientists because data collection is often more accessible than experimental research. These studies, however, do not suggest causative agents within the environment or eliminate competing explanations for observed environmental traits. Causation is difficult to demonstrate within the built environment because “causal pathways in public health are complex and often not fully described” (p. 753) [25], owing to the unpredictable nature of human behavior. Given the lack of causative explanations, there is a need to further evaluate the cause of these indicators of poor health.

Because purely scientific experiments are difficult to conduct in the social environment, natural experiments have been advocated as a proxy method [26]. A natural experiment is a quasi-experimental type of study which takes advantage of an intervention which occurs without researcher control [25]. But the difficulty in forecasting interventions in time to conduct preliminary assessments often stymies the use of this method. 

Natural experiments have been used in various research projects on the built environment. For instance, Fitzhugh and colleagues found a significant increase in total physical activity after the improvement of an urban pedestrian greenway [27]. MacDonald and colleagues found that the addition of a light rail line was associated with reductions in BMI and probability of obesity [28]. Kapinos and Yakusheva, meanwhile, found that dormitory assignment among college freshman was associated with weight-gain and weight-related behaviors. Each of these research findings demonstrates the influence that local environmental changes can have on the health-related behaviors of individuals [29].

Despite the call for health researchers to develop more innovative study designs, few studies to date have utilized natural experiments to attempt to isolate causal links between the food environment and health outcomes. Two natural experiments in Leeds and Glasgow, UK, have found conflicting results regarding the impact of new food retail establishments in socioeconomically disadvantaged regions [30,31]. A recent US study also lacked definitive results that an intervention had a measurable impact on diet [32]. Donald recently noted that the “food desert problem will not be advanced without a large-scale effort to replicate the kind of critical evidence-based ‘before/after’ assessments that Wrigley and his UK researchers did in the 1990s and early 2000s” (p. 2) [21].

The present research, therefore, advances this literature by following a natural experiment to evaluate the impact of a new food retailer on the *food consumption and security* of residents in a socioeconomically disadvantaged neighborhood of Flint, Michigan. In this case, food security refers to whether a respondent has self-reported an instance of lacking food of sufficient quality or quantity within the last year [33]. This is important because when food security is threatened, the consumption of healthy, fresh foods such as vegetables and fruits—and with it, nutrient intake—declines [34,35]. The addition of a new grocery store, therefore, may help food insecure residents reach healthy food more easily by providing an affordable option within a short distance.

As discussed, only three research teams have successfully evaluated natural experiments on food retail interventions, and only the most recent was in the US [30,31,32]. The present manuscript, then, will expand on this rarely studied strain of research and contribute the second article to the American context, which is important because of differing cultural attitudes toward urban development. Strict planning policy in the UK has enabled cities to retain a denser urban fabric than in the US [36], which facilitates active travel and lower obesity rates [37]. The differences in development patterns and cultural attitudes may also contribute to differences in the influence of neighborhood food shopping on consumption behaviors. It is hypothesized that in a culture where citizens use active travel less frequently, immediate neighborhood shopping opportunities will play a weaker role in shaping diet and, therefore, the addition of a new food retail establishment in a car-dependent community will not have a significant effect on consumption habits. The effect on food security may, however, be stronger, if food insecure respondents up-take the new food retail establishment.

## 2. Experimental Section

### 2.1. Study Area

The study area, Flint, Michigan, is a city which has experienced tremendous changes over the past four decades, beginning with disinvestment from car manufacturers in the 1970s. Manufacturing employment has dropped 77% since 1980, contributing to a 41% decline in employment overall [38]. The 1960 Master Plan estimated Flint’s population would soon surpass 400,000 [39]; the US Census Bureau, however, now estimates the population at 102,000 [40]. A decline in services and retailers such as grocery stores accompanied this population loss. Many neighborhoods in Flint exhibit low densities and near complete abandonment by retail and commercial uses. Where retail does exist, grocery stores are easily outnumbered by liquor stores offering junk food and stocking little fresh produce.

In 2009, private investors announced the opening of an independent grocery store (Witherbee’s Market) at the center of the Carriage Town neighborhood, following a broader neighborhood trend in redevelopment from the public and private sectors. Initially, very few residents in Carriage Town could walk to a grocery store, and low-mobility residents would have experienced difficulty in accessing healthy food, but the opening of Witherbee’s nullified this geographic disparity [41]. From a research and public health standpoint, the impending change in the food retail landscape presented an opportunity to evaluate a natural experiment and determine whether the opening of the market would influence residents’ consumption habits or food security.

The evaluation of this intervention on food consumption is important because Flint is one of the least healthy cities in the state of Michigan [42]. Overall, 82% of Genesee County residents do not consume an adequate amount of fruits and vegetables, compared to 78% statewide [43]. Flint residents consume between 3.6 and 4 servings of fruits and vegetables per day (n = 766 and 687, respectively) [43,44], placing them well below the long-suggested 5 servings per day [45]. More troublesome, these surveys estimated that between 68 and 71% of residents were overweight or obese. Within the study neighborhoods for this research, these statistics are assumed to be worse, since poverty and unemployment are higher than the county average. It is valuable to consider this past research in the Flint Metropolitan Area so comparisons may be drawn on the two study neighborhoods.

**Figure 1:**
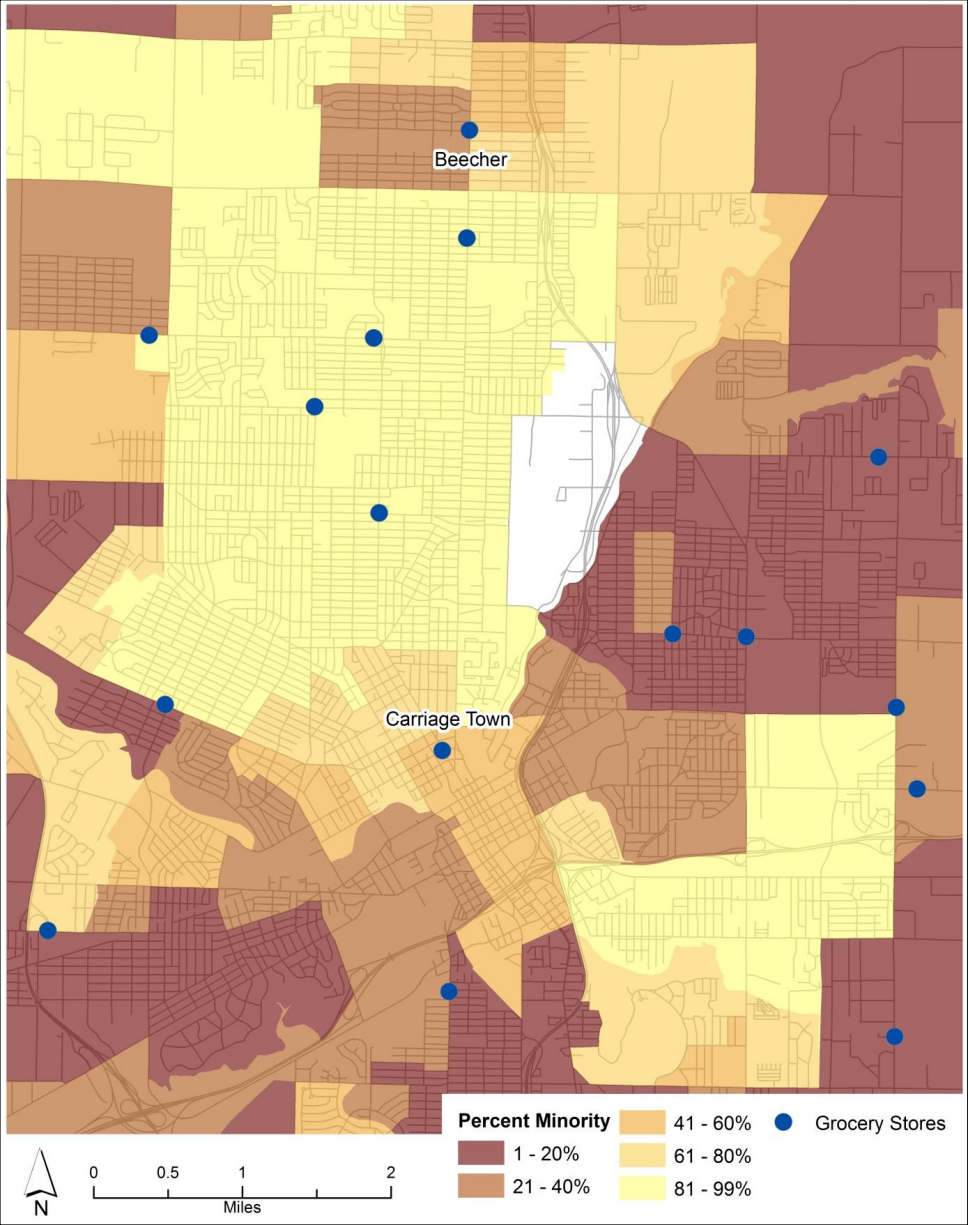
Percent of Minority Residents by Census Block Group (US Census Bureau, 2010).

**Figure 2:**
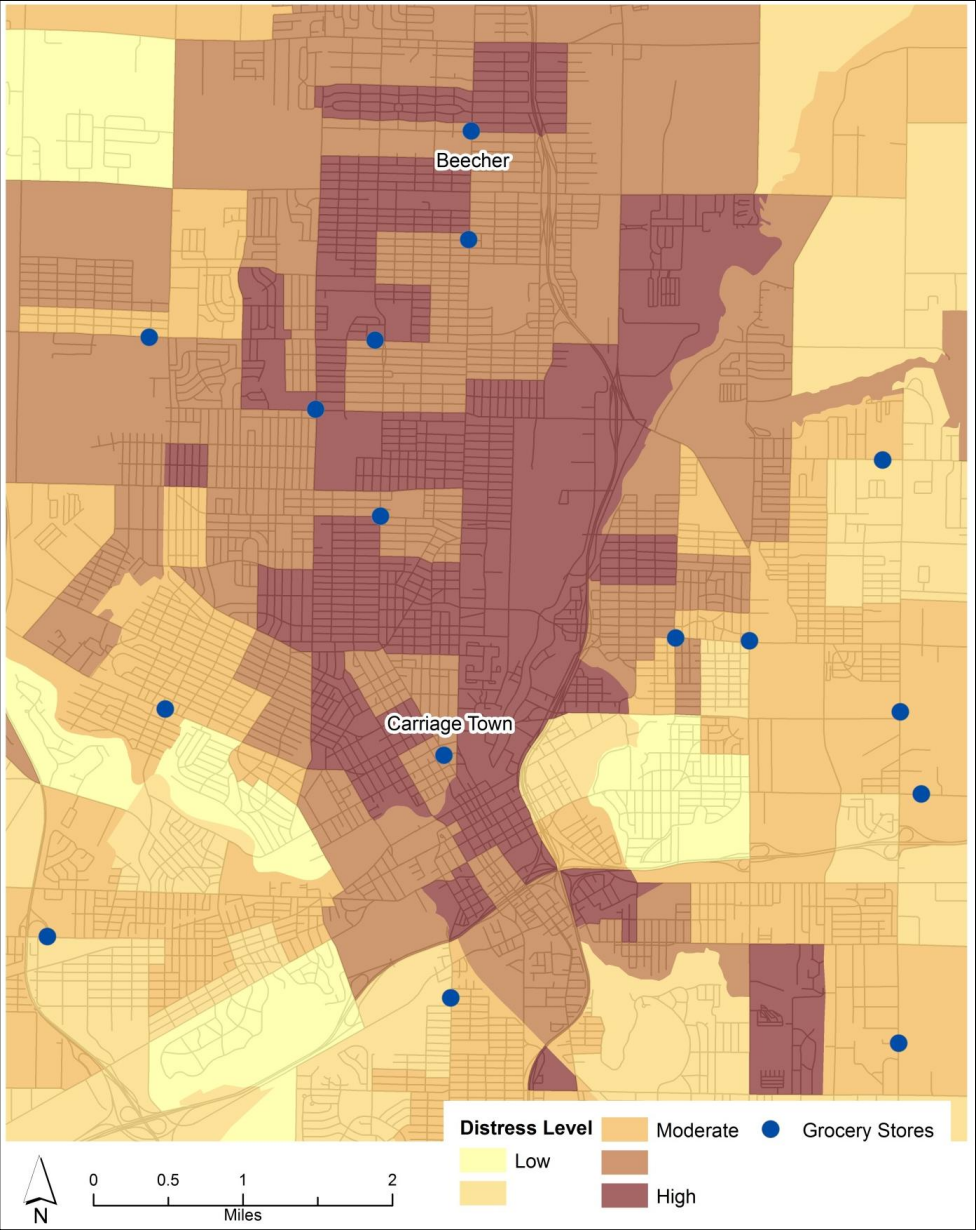
Level of Socioeconomic Distress by Census Block Group (US Census Bureau, 2000).

Site selection of the control neighborhood was constrained by three factors: the size of the metropolitan area, racial segregation, and socioeconomic disadvantage. First, the intervention neighborhood (Carriage Town) lies in the center of the city. The small geographic size of the Flint region created the potential for cross-contamination between control and intervention neighborhoods, leaving few suitable candidate neighborhoods. Previous natural experiments were conducted in larger cities, meaning that intervention and control groups could be placed to avoid cross-contamination [30,31,32]. Second, Flint is a highly racially segregated city, so few neighborhoods were similar to Carriage Town in ethnic composition (Figure 1). Carriage Town is one of only a few neighborhoods where the population is composed of less than 80% either white or black residents. Third, Flint is highly bifurcated socioeconomically. Using a distress index developed in past research [41,46], various socioeconomic characteristics were combined into an index to predict areas of high distress (Figure 2). These characteristics included: incidence of lone parenthood, incidence of low income, educational attainment, and unemployment rate.

After an evaluation of census data for the city and out-county area [47] and the information from Figure 1, Figure 2, the Beecher district on the north side of the urban area was selected because it was similar to Carriage Town in sociodemographic characteristics, the degree of racial integration, and population density. In contrast to Carriage Town, however, Beecher has been served by a grocery store since well before the study began. The presence of a high proportion of black residents in both of these study neighborhoods is an important consideration because past research from Philadelphia showed that healthy food is more difficult to find in neighborhoods with a high proportion of black residents [48]. These neighborhoods may therefore yield populations with issues related to accessing healthy food.

### 2.2. Methods

Data collection spanned two phases: phase one was conducted April-June 2009, prior to the opening of Witherbee’s, and phase two was completed April-June 2011. Witherbee’s opened in June 2010 and closed November 2011, so residents had one year to independently ‘uptake’ the new food source. One year is considered an adequate time period over which to observe behavioral change [49].

Pre-tested questions adapted from the Behavioral Risk Factor Surveillance System (BRFSS) [50] were used in a telephone survey of randomly selected residents in the control and intervention neighborhoods (Beecher and Carriage Town, respectively). Questions covered topics such as: food consumption, food security, shopping habits, motor vehicle availability, and sociodemographic characteristics. Food consumption was measured through the ‘fruit and vegetable’ and ‘nutrition’ BRFSS modules, while food security was measured through the six question ‘food security’ module. Despite varying results of over- and under-estimation of food consumption found in past research when using brief screeners such as these [51], this module was used for the sake of comparability to past community surveys.

Respondents were also asked to indicate the street intersection nearest to their home. All respondents were at least 18 years old and were the primary shopper for their household. Respondent selection was determined by the proximity of the resident to the center of the neighborhood. In both neighborhoods the grocery stores were operationalized as the center of the neighborhood (see in Figure 1, Figure 2).

A randomized sample of phone numbers were taken from within walking distance of the grocery stores (starting within 1,000 m and subsequently including residences within 1,500 and 2,000 m of the store sites). Based on spatial analysis, the population of each neighborhood was estimated to be around 3,000. Supposing a household size of 2.51 [40], 1,200 households existed in each neighborhood. A sample of households with landline telephones was attempted, but due to a high rate of disconnected phone numbers, high rates of respondent refusal in each neighborhood, and high rates of residential vacancy, the sample collected yielded a 15% response rate. In the first phase, 186 responses were collected, while 166 were collected in the second phase, returning a 7–8% sample size. Nevertheless, these values are still considered statistically significant for the neighborhoods under study. As well, comparability between neighborhoods is considered more important than generalizability overall for the purpose of this study.

Because of high residential turnover rates in Flint [52], repeating the study with the same respondents would have been infeasible. Respondents in the second phase, therefore, were asked where they primarily shopped for groceries and, if they had lived in the neighborhood prior to 2010, where they shopped for groceries at that time. This technique controlled for any variation in shopping habits due to drawing from two different samples of the same population. The respondents are thus also treated as four distinct groups as opposed to two groups studied longitudinally (Beecher 2009, Beecher 2011, Carriage Town 2009, and Carriage Town 2011), and statistical analysis reflects this consideration. It was hypothesized that, given the small scale of the store and the many countervailing forces such as entrenched behavioral and cultural practices, dietary habits would not improve significantly due to the addition of the store.

## 3. Results and Discussion

Descriptive statistics were compiled for various predictor and outcome variables and compared to past community surveys (shown in Table 1). Statistics are broken down by phase, neighborhood, and food security status. In general, the sample collected for this research was older, was less educated, comprised more black residents, and consumed fewer fruits and vegetables than the samples from past research [43,44]. Respondents from the intervention neighborhood (Carriage Town) did not vary significantly from respondents in the control neighborhood (Beecher) in food consumption or self-reported health. Additionally, no significant differences were seen in descriptive statistics when considering both neighborhoods pre- and post-intervention.

**Table 1:** Descriptive statistics of present research and past community surveys.

	Average Age	% Female Respondents	% Black Respondents	% No High School Diploma	% Income < $20,000	% Food Insecure	Servings of Fruits & Veg / Day	% Adequate F&V Consumption	% Overweight or Obese	Average BMI	% Excellent or Very Good Health
Alaimo (2008) (n = 766)	44	52	49	13	n/a	n/a	4.0	20	68	n/a	38
STYH CS (2009) (n = 736)	47	73	53	n/a	67	n/a	4.0	18	71	29.3	37
Phase 1 - 2009 (n = 186)	56	73	60	23	75	32	2.5	7	72	29.5	32
Phase 2 - 2011 (n = 166)	53	62	61	17	60	37	2.8	9	72	29.5	40
Carriage Town 2009 (n = 100)	56	65	65	18	65	27	2.6	10	69	29.5	34
Carriage Town 2011 (n = 96)	52	49	61	13	54	39	2.6	5	65	28.5	46
Beecher 2009 (n = 86)	57	82	56	28	85	36	2.5	3	74	29.5	30
Beecher 2011 (n = 70)	57	77	61	24	70	34	2.9	14	82	31.1	31
Food Secure (n = 226)	57	67	51	19	65	n/a	2.6	9	70	29.5	43
Food Insecure (n = 118)	50	69	63	22	82	n/a	2.6	7	75	29.5	25
Total	55	67	61	20	67	34	2.8	8	72	29.5	37

### 3.1. Regression Analysis

The potential for evaluating many relationships (especially with regard to food security, food consumption, and geographic access to nutritious foods) necessitated further analysis. Bivariate regression analysis was run for all predictor and outcome variables to determine the relevance of multivariate regression analysis for fruit and vegetable consumption or food security as outcome variables. Several risk factors were evaluated as shown in Table 2, and significant results are shown with asterisks. Geographic variables referring to the distance from a respondent’s home to the nearest grocery store (SM) or source of nutritious food (NF) were not significant with fruit and vegetable consumption (TFV). Food security (FS) was negatively correlated with both, suggesting that food insecure respondents were living *nearer* to both types of food outlets. The absence of multiple non-related significant relationships to either fruit and vegetable consumption or food security, however, nullified the utility of running multivariate analysis.

**Table 2:** Correlation matrix for all survey variables.

	Fruit & Vegetable Consumption	Food Security	Car Ownership	Self-Reported Health	Gender	Educational Attainment	Household Income
	TFV	FS	MV	HLTH	GEND	EDU	INC
TFV	1						
FS	−0.012	1					
MV	−0.081	−0.128	1				
HLTH	−0.102	0.248^**^	−0.208^**^	1			
GEND	0.082	0.019	−0.091	0.052	1		
EDU	0.125^*^	−0.074	0.160^*^	−0.208^**^	0.019	1	
INC	0.129^*^	−0.143^*^	0.048	−0.219^**^	−0.068	0.311^**^	1
BMI	−0.095	0.000	−0.201^*^	0.270^**^	0.041	−0.034	−0.043
SM	0.008	−0.131^*^	0.235^**^	0.042	−0.074	0.097	0.045
NF	0.052	−0.120^*^	0.173^*^	0.004	−0.078	0.126^*^	0.162^**^
CON1	−0.025	0.018	0.014	−0.076	−0.064	0.170^**^	0.174^**^
CON2	−0.086	0.034	0.023	−0.019	−0.071	0.043	0.003
CON3	−0.072	0.040	−0.005	−0.158^**^	−0.181^**^	0.021	0.185^**^
IMP	−0.247^**^	0.047	0.022	0.318^**^	−0.122^*^	−.184^**^	−0.168^**^
Age	−0.054	0.174^**^	0.157^*^	−0.057	−0.109^*^	0.260^**^	0.097
	BMI	SM	NF	CON1	CON2	CON3	IMP
BMI	1						
SM	−0.009	1					
NF	0.003	0.624^**^	1				
CON1	−0.086	−0.060	-0.036	1			
CON2	−0.02	−0.168^**^	-0.129^*^	0.319^**^	1		
CON3	−0.084	−0.113^*^	-0.124^*^	0.233^**^	0.200^**^	1	
IMP	0.137^*^	0.013	-0.018	−0.213^**^	-0.108^*^	-0.144^**^	1
Age	−0.015	0.006	-0.011	0.077	0.203^**^	0.098	−0.059
* Correlation is significant at the .05 level (2-tailed)							
** Correlation is significant at the .01 level (2-tailed)							

Still, other variables were highly correlated to one another. Echoing prevailing health research, self-reported good health was positively but not strongly associated with higher food security, higher educational attainment, higher income, lower BMI, and stronger feelings about the importance of healthy foods. Various other relationships were significant, but because multiple regression would not yield useful information for the primary outcome variables of food consumption and food security, analysis turned to additional statistical procedures.

ANOVA statistical tests were run to determine whether differences existed in food consumption or food security by neighborhood. Because the respondents varied from phase 1 to phase 2, the before/after groups in each neighborhood are treated as separate populations; thus, the respondents can be split into four groups. If the 2011 Carriage Town group differed from the others, therefore, it would support the notion that this group may have been affected by the intervention. Included in this analysis were geographic variables for access to grocery stores and nutritious foods. Additionally, t-tests were run with dichotomous variables including food security to further uncover relationships between variables and to direct the manuscript toward its primary research aim of answering whether access to a new grocery store would significantly impact food consumption or food security.

### 3.2. Food Security

Studying food insecure respondents as a group is important because 34% of all respondents were food insecure, and two in three respondents had annual incomes of less than $20,000. In contrast, earlier research found that just 21% of city residents and 12% of out-of-county residents had some degree of difficulty accessing healthy food [43]. From Figure 2 and the various descriptive statistics in Table 1, it is clear that the study neighborhoods suffer from multiple levels of socioeconomic disadvantage when compared to the rest of the Flint region. Revealing whether and how food insecure respondents vary from food secure respondents will help in documenting the impact of the addition of a grocery store in one of the region’s most distressed neighborhoods. 

Based on the results of t-tests, food insecure respondents reported poorer health status and were younger. Food insecure respondents did, however, live significantly closer to nutritious food sources. Further descriptive statistical analysis found that 15% of the food insecure did not have access to a car, and an additional 49% had only one working vehicle, compared to 2 and 36% of food secure respondents, respectively. Regarding constraints on mobility, 16% of the food insecure shopped at the nearest grocery store, compared to 7% of the food secure. As shown, food insecurity conveys related characteristics of socioeconomic disadvantage, lack of access to transportation, and close proximity to grocery stores.

### 3.3. Food Consumption

Post-hoc Tukey’s tests were conducted after ANOVA tests to determine how the study neighborhoods differed in terms of food consumption. The 2011 cohort of Carriage Town residents was significantly more likely to eat at restaurants and purchase (typically unhealthy) prepared meals from grocery stores than their counterparts from 2009. Respondents in both 2011 neighborhoods were also statistically more likely than their 2009 counterparts to visit fast food restaurants than they were in 2009. No other differences existed between the two 2009 groups, nor between the two 2011 groups.

**Figure 3:**
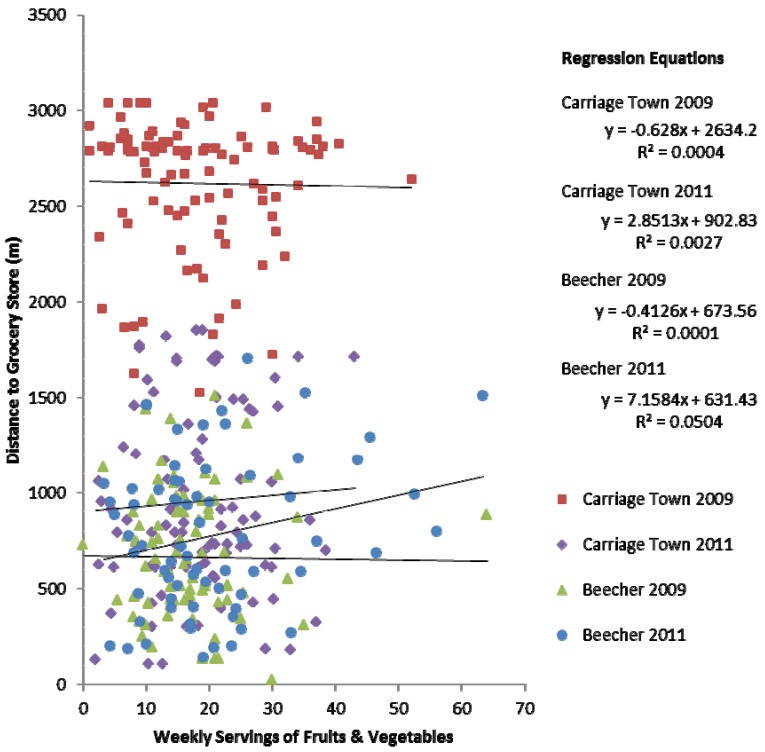
Regression lines for relationships between fruit and vegetable consumption and distance to a grocery store by neighborhood

Tukey’s tests confirmed the notion that Carriage Town 2009 was significantly different from the other three neighborhoods. Yet regression results from Table 2 suggest that geographic distance to grocery stores or healthy food generally did not play a significant role in fruit and vegetable consumption overall. To determine if neighborhood-specific differences existed, therefore, regression analysis was run for various types of food stores and different measurements of consumption, which allowed for comparison of regression coefficients. One notable result is shown as a scatterplot in Figure 3, showing weekly consumption of fruits and vegetables with the distance to the nearest grocery store. As revealed in the regression equations for each neighborhood, there are no significant relationships between fruit and vegetable consumption and proximity to grocery stores at the neighborhood level. 

This null relationship is consistent when evaluating proximity to fast food and convenience stores, and when evaluating the proximity to the second and third nearest grocery stores [53]. The null relationship holds for all neighborhoods individually, and for all variations on consumption (e.g., when considering only consumption of green salads, fruit juice, *etc*.). The null relationship is also consistent when evaluating the relationship between proximity to fast food and the frequency of fast food consumption. The suite of null results suggests that other confounders in the physical or social environment may inhibit dietary habits.

### 3.4. Store Switching and Mobility

Questions from the 2011 sample are also useful for examining whether Carriage Town residents were ‘up-taking’ Witherbee’s and, in general, whether residents in either neighborhood shopped at the nearest store. Spatial analysis was conducted by calculating the network distance from each respondent’s home to their nearest grocery store and validating whether this was their primary grocery store.

In 2009, 10% of Beecher respondents and 1% of Carriage Town respondents shopped at their nearest grocery store. In 2011, 10% from both neighborhoods shopped primarily at the nearest store. Thus while the control neighborhood remained virtually unchanged, 10% of Carriage Town respondents began shopping at Witherbee’s. Additional data on mobility reflects these shopping patterns and suggests that most people are not constrained to shopping within their neighborhood: (1) 93% of households own a private automobile, and (2) only 15% of respondents walk to a store more than once a week. The general lack of active travel and the ubiquity of private transportation suggest that most people are bypassing the nearest store to reach more distant chain supermarkets in the suburbs: 83% of respondents indicated a chain grocery store as their primary food shopping destination.

### 3.5. Discussion

This research addressed the impact of a new grocery store in a former ‘food desert’ on consumption habits, as well as specific characteristics of food insecure respondents. Overall, 92% of respondents did not consume an adequate amount of fruits and vegetables [18], suggesting that their overall dietary quality may be sub-optimal for good health. Given similar findings in past Flint research, issues with healthy eating seem pervasive city-wide, especially in socioeconomically distressed areas. Recognizing the public health issues of unhealthy eating, city officials have supported local-level policies to increase the availability of nutritious foods by facilitating the development of grocery stores and encouraging convenience stores to increase stocks of fresh produce, mirroring efforts in New York to facilitate healthy eating [54].

Within the sample, 34% of respondents were food insecure, though these respondents tend to live closer to grocery stores. Furthermore, more than twice as many food insecure respondents shopped at their nearest grocery store. These facts may reflect a coping mechanism that some low-income, food insecure households employ by living nearer to food sources. This may be a positive starting point, since geographic access is less of an issue for these food insecure residents.

Additional results from this research suggest that geographic interventions lack efficacy in the absence of broader changes to the food environment. No relationship was found between proximity to nutritious food and diet supporting the claim by Coveney and O’Dwyer that “living in a food desert *per se* was not in itself a major misfortune” (p. 48) [24]. The ability to make travel arrangements to nutritious food, for instance, contributed more to issues of food insecurity or poor diet. And indeed, because most respondents in *this* study had access to a car, barriers to accessing food were diminished.

No significant differences were seen in the consumption of nutritious foods in the intervention group (2011 Carriage Town) *versus* the other three groups (2009 and 2011 Beecher, 2009 Carriage Town), suggesting that neighborhood differences did not mediate this type of consumption. The sample *overall* exhibited significantly poorer consumption habits than those of past surveys. This difference is likely because the study neighborhoods are two of the most distressed in the county (both are in the bottom quintile of neighborhood distress in Figure 2), and thus the residents exhibit additional risk factors for consuming poor diets.

Regarding consumption of other foods, there was a significant increase in the purchasing of prepared food within the intervention neighborhood, possibly due to the presence of a deli within Witherbee’s Market. This statistic is supported by informal discussions undertaken with Carriage Town residents, many of whom indicated shopping at the store for sundry items or deli products, or not shopping at the store at all. Rather than facilitating nutritious food consumption, the grocery store may have actually promoted unhealthy eating, since prepared meals became easier to find.

Fast food consumption was also higher among both 2011 groups. Some have suggested that people experiencing economic hardship visit fast food more frequently [55]. The increase, then, may be due in part to an increase in the severity of the economic recession in Michigan from 2009 to 2011. Regardless, the increase in the procurement of unhealthy foods in the intervention neighborhood (both fast food and prepared meals) supports the assertion that Witherbee’s did not have the intended effect of increasing nutritious food consumption.

There was an increase in the % of Carriage Town residents who shopped at their nearest grocer, from 1 to 10%. By comparison, Wrigley and colleagues found significant changes in the number of residents who switched their primary shopping source to a new grocery store [30]. In their research, 45% of respondents shopped at the new store, while 35% claimed the new store was their primary source for fruits and vegetables. The low number of store-switchers in the present research raises questions of economic viability, and likely contributed to the closure of Witherbee’s in November 2011. A preliminary investor-backed financial feasibility report indicated that the store would need to capture 4.3% of the leakage in the neighborhood to remain profitable [56]. Even if the 10% value reflected an overall shift in shopping habits of Carriage Town residents, it is likely most residents were not shopping exclusively at Witherbee’s, because low sales and an inability to pay rent were cited as the reasons for closure in the fall of 2011, along with concomitant troubles creating a marketing campaign [57]. Although the store did not have a positive impact on consumption and ultimately closed, the store may still serve as a cautionary tale for further initiatives to ‘re-store’ food deserts elsewhere.

The lack of efficacy of this food retail intervention demonstrates two interrelated issues: (1) simply providing a new source of nutritious food is not enough to alter entrenched behaviors, and (2) the challenge of improving individual dietary habits must simultaneously consider other biological, environmental, and behavioral variables. Especially in distressed neighborhoods, many socioeconomic and cultural factors may inhibit uptake. Specific to Witherbee’s, a number of reasons for the lack of efficacy may be presented: (1) a glut of unhealthy food options in the neighborhood, including the new store; (2) a lack of marketing by Witherbee’s; (3) the single-tiered approach to altering consumption habits; (4) existing shopping behaviors at other stores; and (5) the relatively higher price of groceries at Witherbee’s [41].

The results of this research also suggest that the issue of unhealthy food consumption pervades more deeply than the standard USDA definition of food deserts might suggest. While one neighborhood was characterized as a food desert through careful assessment, both neighborhoods exhibited poor dietary habits. Indeed, based on previous community surveys, the entire Flint community exhibits many issues related to malnutrition. Thus, the issue of so-called ‘food deserts’ may eschew any micro-geographical definition, and instead be more influenced by meso- or macro-level influences.

Given the abundance of unhealthy food in Flint as in other communities, some researchers are now suggesting that the traditional approach to altering consumption behavior may no longer work [2,58]. Wrigley and colleagues discussed that although improving this form of access may be enough to change the shopping or dietary habits of *some* residents, for many others the constraints fall under economic or behavioral issues [30]. Merely changing where a person shops does not equate with an improved ability to purchase food already perceived to be expensive, nor does it equate with a shift toward choices contributing to a healthier diet.

This paper mirrors the findings of Cummins and colleagues [31], who found no improvement of consumption behaviors in an intervention neighborhood in Glasgow, UK, even after adjusting for external confounding variables. If the results of the present study are repeated in other US-based studies and gain a voice in national food policy discourse, then future efforts may shift away from re-storing initiatives and back toward higher-level interventions such as educational programs, ‘fat taxes’, and food labeling [58]. Behavioral economists also now promote libertarian paternalism as a way to ‘nudge’ people into good behaviors without taking away individual choice [59]. Other researchers emphasize the influence of more local educational and promotional campaigns to encourage healthy eating [60].

For the Flint context, the potential to create educational and promotional campaigns within the neighborhood is complicated by the closure of Witherbee’s Market in November 2011. This reflects a failure on the part of the investors to re-capture even a small percentage of the neighborhood leakage. While the store did not have a significant impact on the neighborhood in terms of diet, it did provide nutritious food as an option. Since Carriage Town once again lacks a grocery store, campaigns to encourage the purchase of nutritious foods are made even more difficult.

### 3.6. Research Limitations

Despite the predictive power of natural experiments, this method has its own limitations. In this study, the inability to re-contact the same respondents in both time periods means that direct longitudinal inferences cannot be made. While assumptions can be made about the comparability of data in each time period, an ideal study would track identical residents in multiple time periods to ascertain the immediate and long-term effects of the intervention. Even so, issues of population migration and neighborhood self-selection can skew results; as Lytle indicates, it is difficult to separate contextual and endogenous factors [5]. Furthermore, and as expected, the sample for this study was small and limited to two highly distressed neighborhoods. As a result, this study is not generalizable to the city as a whole. Still, the concentration of respondents in two areas is intended to aid comparability between the two study neighborhoods.

Schafer-Elinder and Jannson are cautious of relying purely on quantitative evaluations of natural experiments, saying “proof of causality...can only be established through controlled and randomised intervention trials, which are almost unthinkable when it comes to wider environmental factors” (p. 309) [61]. Cummins and colleagues also mention that natural experiments “cannot easily disentangle the effect of the hypermarket from other known or unknown interventions” (p. 1040) [31]. Nevertheless, Petticrew and colleagues suggest that natural experiments will remain the best way for social scientists to collect evidence on the possible socio-spatial determinants of health inequalities [25].

## 4. Conclusions

The present research advances the knowledge of food retail-led interventions in North America and contributes to the discourse on questioning the meaningfulness of ‘food deserts’ and accompanying access-driven interventions in a geographic sense. The findings provide evidence that consumption habits of residents in the intervention neighborhood were unchanged by Witherbee’s. Further questions are raised concerning what interventions *would* be the best to help Flint residents, especially after the store’s closure. As demonstrated, the respondents did not eat healthy and food insecurity was an issue for many—just 8% consumed an adequate amount of fruits and vegetables, 72% reported being overweight or obese, and 34% were food insecure. Given the complex nature of dietary interventions, and the recognition that food insecurity cannot be remedied by a single-focus approach, some type of multi-pronged intervention is needed [62,63].

Fortunately for Flint, other programs are underway which aim to combat unhealthy eating and increase access to nutritious food. The Edible Flint collaborative promotes community gardening and healthy eating as strategies for local empowerment and economic development [64]. One of the organization’s work groups recently released a report on the price of nutritious foods in convenience stores, with a goal to use the information to create their own intervention [64]. Organizations such as the Flint Farmers’ Market and local urban farmers also advocate healthy eating as a way to bolster the local economy [65]. Given the evidence from this study and anecdotal evidence from the community, these groups are planning programs beyond retail-led interventions. In concert with programs to get people involved in the food system, programs of a behavioral nature may be able entice consumers to eat more healthy foods.

But further natural experiments of food retail-led interventions are also necessary, since communities exhibit varying characteristics regarding dietary habits and health, and the built environment is not likely to influence each community equally. In Flint, the birthplace of General Motors, the proliferation of the automobile may serve to ease the friction of distance for many people, while simultaneously creating a lifestyle which contributes to unhealthy habits and obesity. While the relationship between the built environment and dietary habits may vary by community, continuing to seek appropriate dietary interventions remains a critical need to address public health issues related to malnutrition.
